# Evolutionary cell biology comes of age

**DOI:** 10.1242/jcs.264348

**Published:** 2025-12-19

**Authors:** Gautam Dey, Lillian Fritz-Laylin, Snezhana Oliferenko, Eelco C. Tromer

**Affiliations:** ^1^ Cell Biology and Biophysics, European Molecular Biology Laboratory, 69117 Heidelberg, Germany; ^2^ Howard Hughes Medical Institute and the Department of Biology, University of Massachusetts, Amherst, MA 01003, USA; ^3^ Randall Centre for Cell and Molecular Biophysics, School of Basic and Medical Biosciences, King's College London, London, SE1 1UL, UK; ^4^ The Francis Crick Institute, 1 Midland Road, London, NW1 1AT, UK; ^5^ Cell Biochemistry, Groningen Biomolecular Sciences and Biotechnology Institute, Faculty of Science and Engineering, University of Groningen, Nijenborgh 7, 9747 AG Groningen, the Netherlands

**Keywords:** Evolutionary cell biology, Comparative cell biology, Phylogenomics, Evolutionary theory, Emerging model organisms

## Abstract

Evolutionary cell biology is emerging as a vibrant discipline, integrating comparative cell biology, evolutionary theory and modern molecular approaches to understand how cells evolve and diversify. With roots dating back to the foundational work of Darwin and Haeckel in the 1800s, the field was historically eclipsed by a focus on a handful of genetically tractable model organisms. Yet, breakthroughs in genomics, imaging, experimental evolution and phylogenetics are driving the rapid growth of the field. Modern evolutionary cell biology faces four central challenges: integrating cell biology with evolutionary theory and experimental evolution to understand both adaptive and non-adaptive processes, bridging the genotype–phenotype gap, identifying and developing new model systems beyond traditional organisms to capture the full diversity of cellular mechanisms, and integrating ecological context with evolutionary processes to understand how environmental forces shape cellular phenotypes. In this Perspective, we discuss how meeting these challenges will illuminate fundamental evolutionary rules governing cellular complexity, innovation and adaptation across the tree of life, with potential applications for predicting cellular responses to future environmental challenges.

## Teaser

**Summary:** This Perspective discusses how the discipline of evolutionary cell biology, by integrating evolutionary theory, comparative physiology and modern molecular approaches, works to understand how cells evolve and diversify.

## Introduction – the deep roots of evolutionary cell biology

Although ‘evolutionary cell biology’ as a term has only been used for the past 20 or so years, the roots of the field reach as deep as modern biology itself. The field of evolutionary cell biology integrates comparative cell biology with molecular evolution to understand how cells evolve and diversify. As a result of a series of scientific expeditions and the founding of the first marine stations in the 1800s (Challenger Expedition, 1872–1876; Ostend founded in 1843; Stazione Anton Dohrn in 1872; Egerton, 2014), the true extent of cellular biodiversity was becoming apparent at the same time that the foundational theories of evolutionary biology were being formulated and debated (Darwin's Origin of Species, 1859; Haeckel's Stettiner Vortrag, 1863). It was natural that these two nascent fields were to heavily influence each other, a phenomenon illustrated by the close working relationship between Darwin, who was focused on evolutionary theory, and Haeckel, who was obsessed with biological diversity both at the cellular and organismal levels (Kutschera et al., 2019).

Celebrating 100 years of The Company of Biologists 2025 marks 100 years since the formation of The Company of Biologists. As part of our celebrations, we are sharing content about the past, present and future of the Company and reaching out to extraordinary members of our community to bring you new and original material.

Gautam Dey is a Group Leader at the European Molecular Biology Laboratory (EMBL) in Heidelberg, Germany, where his lab investigates the evolution and diversity of mitosis in microbial eukaryotes. Lillian Fritz-Laylin is a Professor of Biology and HHMI Investigator at the University of Massachusetts, Amherst in the United States. Her lab studies evolution and regulation of the cytoskeleton across eukaryotic phyla. Snezhana Oliferenko is a Professor of Evolutionary Cell Biology at King's College London and a satellite group leader at the Francis Crick Institute, London, UK, where her research focuses on comparative and synthetic approaches to understanding the evolution of membrane-centred cell biological processes using different fission yeast species. Eelco Tromer is Senior Researcher and Dutch Research Council Veni Fellow at the University of Groningen in the Netherlands, where he studies mechanisms and evolutionary origins of chromosome recombination and segregation in microbial eukaryotes.

Gautam, Lillian and Snezhana are members of the Editorial Advisory Board of Journal of Cell Science (JCS) and were co-organisers of the 2024 JCS Journal Meeting on ‘Diversity and Evolution in Cell Biology’. Gautam and Eelco also co-organised The Company of Biologists Workshop on ‘Genotype to Phenotype: Bridging Comparative Genomics and Cell Biology’, held in 2022.

A hundred years later, scientists took the lessons learned from studying evolution on a macroscale and began to explore how cells and their subcellular compartments evolved in the first place (Gray, 2017; Higgs and Lehman, 2015; Miller, 1953). In the absence of a cellular fossil record, much of this work relied on insights drawn from a wave of ultrastructural studies of diverse microorganisms in the first golden age of electron microscopy (Heath, 1980), along with comparative biochemistry and metabolism (Bloch, 1979). The invention of molecular phylogenetics by Woese and colleagues in the late 1970s revolutionised our understanding of evolutionary relationships (Sapp and Fox, 2013). At the same time, the rise of the ‘model system’ paradigm focused much of the work on a handful of cell types and organisms that have been readily amenable to experimental manipulation. Although one could argue that this powerful new paradigm relegated the study of cellular biodiversity to the background, research in model organisms has been indispensable – it established the intellectual framework that allowed us to recognise that complex cell phenotypes can be explained by underlying molecular mechanisms, laid the foundation for our modern understanding of fundamental cell biology and fuelled the development of tools needed to address biological questions mechanistically, including molecular biology and reverse genetics. These studies have laid an essential foundation for the further development of the nascent field of evolutionary cell biology.

It is worth highlighting that, alongside the swing towards ‘model system’-centred research in the late 20th to early 21st century, the quest to understand the diversity of cellular processes at the molecular level was alive and well – particularly in the field of cellular parasitology. Unicellular parasites, with their many specialised adaptations, provide a natural example of evolutionarily related species with significant trait variation. Long before evolutionary cell biology was recognised as a distinct field, parasitologists were already employing its core approaches: examining cellular structures across related species, linking molecular mechanisms to evolutionary processes and revealing how cellular machinery can diverge despite functional constraints. Their studies challenged the assumption that eukaryotic cellular components are universally conserved and demonstrated how cellular machinery can be modified, repurposed or even reinvented to generate novel solutions to common cellular challenges.

Two parasite groups – trypanosomatids and apicomplexans – stand out as early subjects of what has become known as evolutionary cell biology and have provided fundamental insights into the diversity and plasticity of eukaryotic cells. Trypanosomatids, for instance, possess a unique mitochondrial genome organisation known as the kinetoplast, which consists of interlocking circular DNA molecules. This complex structure is thought to have evolved from a simpler ancestral state (Lukes et al., 2002), an early example demonstrating how mitochondrial architecture can be dramatically reshaped by evolutionary pressures. Similarly, studies of apicomplexans using molecular phylogenetics and cell biology approaches led to the discovery of the apicoplast, a non-photosynthetic organelle that originated from a chloroplast acquired through secondary endosymbiosis (an endosymbiont derived from another eukaryote rather than from acquisition of a free-living prokaryote). Over time, the apicoplast shed its photosynthetic capabilities, adapting instead to metabolic functions essential for parasite survival in the intracellular environment (McFadden et al., 1996). Both examples illustrate how parasitic lifestyles drive the evolution of novel cellular traits, offering key insights into the flexibility and innovation of eukaryotic cell biology.

More generally, microbial eukaryotes – with their incredible diversity in cellular form and function – have inspired incisive analyses of the rules underlying cellular organisation and hypotheses for the evolution of cellular features. Trailblazing works by Heath (1980), Raikov (1994), Pickett-Heaps (Pickett-Heaps, 1975, 1999) and others have conceptually expanded our understanding of the eukaryotic nucleus, nucleocytoplasmic domains, microtubule-organising centres, mitotic spindle assembly and nuclear envelope remodelling. Likewise, many evolutionary protistologists have long embraced a broader evolutionary cell biology perspective, using comparative genomics, phylogenomics and ultrastructural analyses across diverse eukaryotic lineages to reveal how cell structures and organelles have diversified and been repurposed over evolutionary time (Richardson et al., 2017). Furthermore, although bacteria and archaea have classically served as powerful models for molecular biology and genetics rather than cell biology, recent advances in imaging, especially super-resolution microscopy techniques, have opened up new avenues of research in these organisms (Gitai, 2005; van Wolferen et al., 2022; Springstein et al., 2025 preprint). Of particular note, a growing appreciation of the ubiquitous roles of condensates in cellular physiology has been accompanied by the realisation that bacterial (Azaldegui et al., 2021) and archaeal (Islas-Morales et al., 2023) cells might be extensively compartmentalised – just not using lipid membranes. These discoveries are just the tip of the iceberg: massive advances in sampling bacterial and archaeal diversity (Liu et al., 2021b) across biomes, particularly in the soil and the deep ocean, have led to landmark discoveries of new biology as well as an increasingly clear understanding of the evolutionary trajectories that gave rise to the three domains of life.

In this Perspective, by building on the rich history of the field, we highlight in subsequent sections the central challenges facing evolutionary cell biologists in the current era, synthesised into a guiding vision for the future.

## A guiding vision for modern evolutionary cell biology

The early 2000s were marked by a growth of interest in integrating concepts from evolutionary and cell biology. This interest, along with an emerging flood of genomic data, set the stage for the coalescence of a new scientific community with explicit goals and language. The National Science Foundation-sponsored 2012 workshop on Evolutionary Cell Biology (https://www.nsf.gov/reports/topical/workshop-evolutionary-cell-biology) was a key step in this process that underscored the importance of combining comparative genomics with insights from population genetics and classic cell biology. The workshop concluded that evolutionary cell biology should aim to explain two main questions. The first is what evolution has produced: to address this, evolutionary cell biologists can use comparative analyses of organisms belonging to different clades to identify which molecular building blocks and cellular structures exist and in what forms. The second is how evolution happens: this can be addressed using population-level data and mechanistic cell biology to understand how new variants emerge and are maintained or lost.

**Fig. 1.**
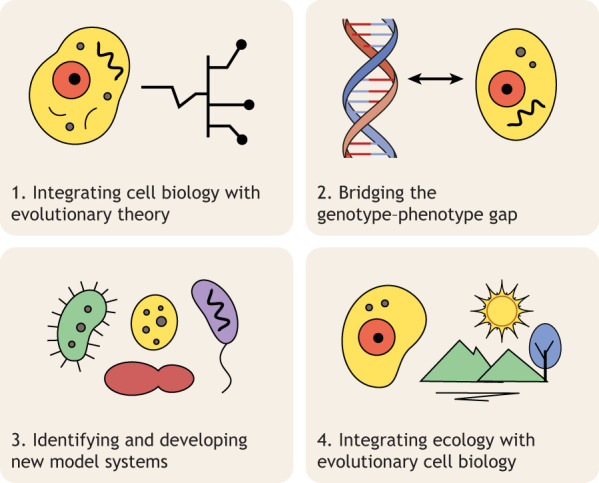
**Key challenges in evolutionary cell biology.** This schematic illustrates four central challenges in the field: (1) integrating cell biology with evolutionary theory and experimental evolution, (2) bridging the genotype–phenotype gap, (3) identifying and developing new model systems, and (4) incorporating ecological context into evolutionary cell biology. Each quadrant highlights a conceptual hurdle that must be addressed to advance the discipline.

This duality – pattern and process – remains at the heart of evolutionary cell biology. This field brings together cell biology, evolutionary theory, genomics, population genetics, biophysics and ecology, not only to catalogue cellular diversity but also to discern the rules and constraints that govern the origins, maintenance and modification of cell biological systems. Achieving this goal requires the field to meet four key challenges (Fig. 1) to establishing causal inferences that tie together the mechanistic underpinnings of cell biology with population-genetic and ecological processes, set against the broader backdrop of Earth's geological history.

## Challenge 1 – integrating cell biology with evolutionary theory and experimental evolution

Comparative cell biology, physiology and genomics offer powerful insights into both conserved and variable mechanisms that underpin cell function, but they cannot explain how organisms arrive at their individual solutions or how key evolutionary transitions occur. To answer these questions, we must link variability in cell phenotypes to underlying genetic variation and determine the evolutionary forces that gave rise to that genetic variation. This is not an easy task. Even in unicellular organisms, where the relationship between cellular phenotype and organismal fitness can be straightforward, cell biology is likely to be ‘optimised’ as a whole, to satisfy the many different – and often conflicting – needs of an organism (Molenaar et al., 2009). Such relationships are subject to a host of additional constraints in multicellular systems, making it even more difficult to link genetic variation to specific phenotypes that evolution can act on. Muddying the waters further, diversification in cellular organisation can be driven both through lineage-specific expansion or simplification of genetic networks (O'Malley et al., 2016). Importantly, evolution occurs at the level of populations, with the non-adaptive processes (such as mutations, recombination, gene flow, genetic drift) providing the fuel for natural selection (Lynch, 2007a,b). To move the field forward, we must adopt the analytical tools of evolutionary biology to study how cellular traits evolve and let go of the default assumption that every feature must be adaptive.

### Disentangling the contribution of adaptive versus non-adaptive events requires evolutionary thinking

Perhaps the most compelling examples of the adaptive potential of newly acquired genetic changes result from horizontal gene transfer (HGT) and other forms of lateral gene flow, such as introgression through interspecies hybridisation (the crossing of two species from the same genus; Gallone et al., 2019; Pryszcz et al., 2015; Soucy et al., 2015; Stairs et al., 2018). HGT-derived phenotypes are often simple and readily portable – that is, encoded by a single gene or an operon of functionally related genes – and require minimal initial connectivity with host biology. Their acquisition endows organisms with novel traits, such as utilisation of new resources (Wybouw et al., 2016), new approaches for defence or predation (Moran et al., 2012), or the ability to thrive in new environments (Boschetti et al., 2012; Rao et al., 2025; Stairs et al., 2018).

Yet, even HGT-dependent evolution involves additional changes that occur as a part of the ‘domestication’ process (Lercher and Pál, 2008), which integrates horizontally transferred genes into the cellular logic of the host. On the evolutionary timescale, it may also rewire the host cell biology. For instance, the domestication of prokaryotic hopanoid synthesis has allowed the fission yeast *Schizosaccharomyces japonicus* to thrive in anoxia, where the oxygen-consuming production of essential membrane sterol lipids is not possible (Bouwknegt et al., 2021; Rao et al., 2025). In turn, accommodating hopanoids alongside sterols might have necessitated a metabolic switch to membranes rich in unusual asymmetrical glycerophospholipids, attenuation of sterol biosynthesis and changes in the organisation of many transmembrane domains, in comparison to other fission yeasts (Makarova et al., 2020; Rao et al., 2025). This case additionally underscores the challenge of gaining evolutionary insights by purely comparative approaches. Far from being ‘adaptive’, profound differences in membrane-associated processes, including mitotic nuclear membrane remodelling, cytokinesis, cell polarity and mitochondrial energetics, between *S. japonicus* and a related fission yeast *Schizosaccharomyces pombe* (Foo et al., 2023; Gu and Oliferenko, 2019; Kaino et al., 2018; Pieper et al., 2020; Russell et al., 2017) might have arisen under global metabolic and biophysical constraints. Indeed, cellular metabolism can constrain the evolutionary processes acting on complex traits (Kempes et al., 2012; Lemke et al., 2025; Pfeiffer et al., 2001; Vitkup et al., 2006). Developing systematic frameworks to quantify and describe non-adaptive evolutionary trajectories will help to identify cellular phenotypes that are operating in an adaptive selective regime while simultaneously reducing the tendency to create ‘just-so’ arguments for every observable phenotypic change.

### Experimental evolution provides tools to study evolutionary processes in real time

Depending on the approach, experimental evolution can estimate mutation rates and determine the genetic basis of phenotypic variability (Halligan and Keightley, 2009); identify possible mechanistic routes to adaptation to changing environments (Long et al., 2015); probe the ‘evolvability’ of cellular functions (Laan et al., 2015; Liu et al., 2015; Rancati et al., 2008) and co-evolution of traits (Jiang and Zhang, 2023); explain how organismal life history, genome organisation and genetic interactions affect adaptation (Selmecki et al., 2015; Sharp et al., 2018; Vande Zande et al., 2023; Verstrepen et al., 2005); and define the requirements for evolutionary rescue (the process by which a population that would have gone extinct is able to persist by natural selection acting on heritable variation; Bell and Gonzalez, 2009; Ramsayer et al., 2013). Beyond fundamental science, experimental evolution frameworks are routinely used in biomedical and biotechnology applications (Hunt et al., 2010; Sandberg et al., 2019).

Adaptive laboratory evolution (ALE) approaches have much to offer in terms of cell biological insight, but they are necessarily limited by trait measurability. It is relatively easy to design ALE experiments, which measure an increase in overall fitness (e.g. growth rate) in adverse conditions or in response to deleterious genetic changes introduced by the investigator. Selecting for physical properties (e.g. sedimentation) may enable the investigator to probe the biology and the evolution of global cellular and supracellular features, such as cell size and cell group dynamics. For instance, a long-term evolutionary experiment with ‘snowflake’ *Saccharomyces cerevisiae*, in which clonal cell groups arise through successive rounds of cytokinesis without separation (Ratcliff et al., 2012), has provided fascinating insights into possible metabolic constraints acting on group size evolution (Bozdag et al., 2023; Wong et al., 2025) and the emergent biophysical properties of cell groups, such as metabolically driven fluid flows (Narayanasamy et al., 2025). Yet, it is considerably more challenging to design an ALE approach acting explicitly on subcellular features. Advances in imaging-assisted cell sorting (Di Trapani et al., 2018; Nedbal et al., 2015; Schraivogel et al., 2022) may allow selection for rare events that affect complex cellular traits, expanding the scope of ALE applications (Helsen et al., 2023). Importantly, laboratory evolution experiments can be used to probe if and how the adaptation to ecologically relevant selection pressures affects cellular-level features.

### Population genetics can probe the evolution of cell biological traits

Each evolutionary scenario – such as whether a specific trait is neutral or adaptive, or how distinct traits relate to each other – can be examined explicitly by measuring within-species variation of traits and their relationship to the fitness of individuals (Lynch and Walsh, 1998). For instance, high-throughput imaging of mitotic spindle dynamics, population genetics and animal fecundity measurements showed that stabilising selection on the size of single-cell embryos in nematodes can explain phenotypic variation in mitotic spindle features. This in turn suggested that spindle traits correlate with cell size rather than themselves evolve under selection (Farhadifar et al., 2015). In another example, investigation of strains of a multicellular cyanobacterium, *Fischerella*, which have diverged in thermotolerance, identified the evolutionary origin of a novel, highly gas-impermeable nitrogen-fixing cell type (Miller et al., 2020). This study also demonstrated that novel traits contributing to ecological specialisation might result from genetic assimilation and adaptive refinement of plastic traits.

Populations of the same species inhabiting different environments can be also directly mined for putative adaptive alleles, with insights relevant to mechanistic cell biology, biotechnology and biosphere response to climate change. Approaches range from identifying genetic targets of selection without prior knowledge of relevant traits (Li et al., 2008), to quantitative trait locus (QTL) mapping to discover genome regions associated with the adaptive phenotype (Liti et al., 2017), to directly assessing the variability in the sequences of candidate genes known to control the trait under investigation. Measuring a cellular trait and understanding its ecological context is particularly powerful when the species is experimentally tractable. As an example, phenotyping growth and branching in isolates of the fungus *Ashbya gossypii* from different climates linked temperature sensitivity to a variability in the sequence of a short intrinsically disordered region in the cell cycle and cell polarity regulator Whi3. The experimental tractability of *A. gossypii* has allowed for a mechanistic explanation underlying this divergence, with data suggesting that sequence changes fine-tune the properties of Whi3 condensates required for its function (Stormo et al., 2024).

Beyond evolutionary insights, stepping outside the paradigm of ‘standard laboratory conditions’ to embrace the natural variability and phenotypic plasticity of model organisms offers an orthogonal approach to study cell biological processes and expands the range of biology to explore.

## Challenge 2 – bridging the genotype–phenotype gap

A fundamental challenge in evolutionary cell biology is to understand how genomic changes (genotype) – ranging from single-nucleotide mutations and gene losses to major genomic innovations, from the emergence of new genes up to whole-genome duplication – translate into specific cellular traits (phenotype). Despite dramatic advances in both genome sequencing of diverse species spanning the tree of life and large-scale gene perturbation screens in established and emerging model systems, we generally remain far from being able to robustly predict cellular phenotypes from genomic sequences alone, but some progress is being made. For instance, general metabolic strategies in prokaryotes can now be inferred based on a genomic footprint (Gralka et al., 2023). However, identifying a set of specific genes that underlie and thus give a window to predict key cellular traits of newly sequenced eukaryotic microbes remains a hard problem to solve. Traits such as mode of locomotion, cellular organisation and shape, trophic modes, prey preferences, the presence of specific cell types or how they respond to different ecological cues are currently not easily predicted from the genome alone (Keeling, 2019). Why does this predictive gap persist, and what progress has been made in addressing this problem in evolutionary cell biology?

From the early days of molecular cell biology, genotype–phenotype mapping has largely relied on classical forward genetics, such as Hartwell's ‘cell division cycle’ screens in budding yeast, which linked single gene mutations to specific defects in cell division (Hartwell et al., 1973). Such studies exemplified the canonical ‘one gene, one function’ paradigm that profoundly shaped genetic thinking, encouraging cell biologists to methodically tag or knock out individual genes in model organisms, often focusing on gene essentiality and clear-cut phenotypes assayed under well-defined laboratory conditions. The arrival of affordable whole-genome sequencing has heralded a dramatic expansion of comparative genomics, allowing for the inference of gene function by tracking gene co-presence and/or co-absence across multiple species, a strategy known as phylogenetic profiling (Ciccarelli et al., 2006; Dey and Meyer, 2015; Eisen, 1998; Koonin et al., 2000). Today, surveying hundreds and thousands of genomes allows us to link conserved cellular traits (e.g. cilia or specialised metabolic pathways) to specific gene sets. Coupling such insights to mechanistic experiments in model organisms has led to identification of novel molecular players in fundamental cellular processes (de Wolf et al., 2021; Dobbelaere et al., 2023; Fritz-Laylin et al., 2017).

Despite the successes of these powerful comparative approaches, four evolutionary phenomena limit their broad application for genotype–phenotype mapping. First, genes are frequently pleiotropic, making it challenging to infer direct causal genotype-to-phenotype relationships (Dudley et al., 2005). Second, sequence similarity does not always imply conserved functions (challenging the ‘orthology conjecture’, which posits that genes descended from a common ancestor – orthologues – tend to retain equivalent functions across species), as domains or short linear motifs can be lost or novel molecular interactions may arise in evolution, causing profiling methods to infer erroneous functional links (Plowman et al., 2019). Third, genetic effects can remain hidden under standard laboratory conditions. For instance, neutral variants that have no immediate fitness consequence in standard laboratory conditions may become pivotal under different ecological circumstances (Kimura, 1983; Stoltzfus, 1999); thus, the impact of knocking out a single gene may depend on the presence or absence of other alleles or regulators (epistasis) (Kondrashov and Kondrashov, 2015; Weinreich et al., 2013), or constraining evolutionary trajectories by historical genetic interactions (entrenchment) (Schulz et al., 2025; Travisano et al., 1995). Finally, convergent evolution complicates straightforward mapping of phenotype to genotype by providing evolutionarily distinct genetic routes to arrive at similar phenotypic solutions, as in the case of prokaryotic and eukaryotic flagella (Beeby et al., 2020).

To overcome these evolutionary hurdles, both the scale and resolution of genotype–phenotype mapping efforts have significantly increased over recent decades due to ongoing technological innovations. At the gene level, genome-wide CRISPR knock-out and CRISPR interference and activation (CRISPRi/a) screens can now test tens of thousands of loci in parallel, exposing context-specific essentiality and dense epistatic networks in a wide variety of species (Costanzo et al., 2010; Li et al., 2019; Sidik et al., 2016). Deep mutational scanning efforts, in which the fitness impacts of all possible amino acid substitutions in one protein are systematically assessed, are becoming affordable (Fowler and Fields, 2014). When combined with ancestral state reconstruction (ASR) – a computational approach that infers ancient gene or protein sequences from contemporary ones – deep mutational scanning can now reveal how historical substitutions have shaped contemporary cellular phenotypes, thereby illuminating the evolutionary genotype–phenotype map of protein sequence function (Hochberg and Thornton, 2017; Schulz et al., 2022; Starr et al., 2018). This information might inform our understanding of cellular evolution, assuming such rules could be applied recursively to higher levels of cellular complexity. Although not yet scalable to all genes in one organism, broad expression QTL (eQTL) mapping approaches in yeast (Nguyen Ba et al., 2022), combined with extensive strain engineering capabilities (Taghon and Strychalski, 2023), might already provide opportunities to perform deep mutational scans for specific phenotypes and help us to resurrect ancestral states, potentially informing us on the rules of cellular evolution.

It is important to integrate such genetic insights with phenotype-informed approaches. At the proteome level, spatial proteomics methods (e.g. hyperplexed localisation of organelle proteins by isotope tagging, HyperLOPIT) could illuminate evolutionary shifts in subcellular organisation, especially when combined with phylogenetic profiling (Barylyuk et al., 2020; Moloney et al., 2023). Massive protein tagging projects in unicellular parasites, such as *Trypanosoma brucei* and *Leishmania mexicana*, have extended such functional analyses beyond a handful of traditional model eukaryotes (Sunter et al., 2023). High-throughput single-cell sorting followed by morphological phenotyping and genome sequencing – such as automated imaging combined with expansion microscopy – now allows large-scale ultrastructural characterisation in diverse microbial eukaryotes at scales that are well suited for phylogenetic profiling efforts (Mikus et al., 2025).

Although epistasis, context dependence and incomplete sampling, when taken together, render a predictive genotype–phenotype map still out of reach, the development of large language model (LLM)-based predictors like AlphaFold (Jumper et al., 2021) suggests that the ever-growing scale of datasets combined with advanced AI-driven computational modelling might help achieve this goal in the near future. Moonshot projects like the Earth BioGenome Project (Lewin et al., 2018) aim to sequence the genomes of all known species, providing a broad platform to sample all present-day genotypic variation, but similar broad-scale phenotyping efforts are currently in their infancy. Beyond morphological phenotyping of cells using advanced imaging-based approaches (Mikus et al., 2025), the next frontier is accurately predicting the activity of a protein purely based on its sequence. Most advances to date have come from commercial developers of AI-based protein design platforms, such as Cradle (e.g. Cradle AI, https://www.cradle.bio/). These emerging AI-driven strategies show great promise for making significant inroads into bridging the genotype–phenotype gap in the future.

## Challenge 3 – identifying and developing new model systems

Historically, biologists selected organisms to study that were tailored to their research question. Walther Flemming, for example, studied salamander cells to understand mitosis because their giant chromosomes were easy to track during cell division (Yanagida, 2014). However, this question-driven approach to model system selection diminished as molecular genetic tools emerged in the 1980s for a small number of species. The ability to dissect mechanisms at the molecular level, coupled with the enormous investments of time and resources necessary to develop genetic tools, drove many cell biologists to work on a few model organisms like mice, flies and yeast. For decades, this resulted in hundreds of laboratories working on a handful of model species and accumulating powerful genetic toolkits that enabled detailed molecular investigations, resulting in a sharp narrowing of studied species. Now, most researchers choose their model system based on tool availability rather than how the organism interfaces with their research question.

Despite their utility, established model systems like *Escherichia coli*, budding yeast and flies cannot address every biological question due to their inherent biological limitations. For example, magnetotaxis can only be studied in species that have the organelles needed to sense magnetic fields (Murat et al., 2010). Even for behaviours that are shared across lineages, there are three compelling reasons to expand beyond the handful of well-established model systems. First, certain species possess unique biological properties that make them exceptionally well suited for investigating specific questions. The discovery of telomerase exemplifies this point – researchers capitalised on the unique ciliate biology of *Tetrahymena*, which maintains thousands of telomeres and consequently expresses high levels of telomerase, facilitating the identification and characterisation of this enzyme (Blackburn, 2009). Second, we now recognise that the molecular mechanisms underpinning similar biological processes substantially differ between lineages. For example, the overall architecture of the mitotic spindle can vary wildly between species (Drechsler and McAinsh, 2012; Shah et al., 2024; Velle et al., 2022; von der Heyde and Hallmann, 2022). Therefore, we need molecular-level insights across diverse systems. Third, understanding the evolution of cellular traits demands comparative approaches across related species. As discussed earlier, comparative studies of parasites like trypanosomatids and apicomplexans with their free-living relatives have revealed the evolutionary origins of unique cellular structures like the kinetoplast and apicoplast.

Moving beyond existing model species will require significant resources and effort, but the timing for diversifying our model systems has never been better: technological advances have dramatically reduced the barriers to developing organism-specific tools, scientists have created inherently more adaptable technologies (e.g. next-generation sequencing technology and CRISPR tools), and decades of experience have taught us how to effectively transfer and adapt tools from established model systems to new systems, including species of bacteria (Caulton and Lovering, 2020), archaea (Auernik et al., 2008; Brown et al., 2025; Pohlschroder et al., 2025; Schocke et al., 2019; Trujillo and Komeili, 2025), protists (Ah-Fong et al., 2018; Booth and King, 2020; Faktorová et al., 2020; Kaur et al., 2018), fungi (Kalinka et al., 2024; Liu et al., 2021a; Wilson and Harrison, 2021) and plants (Belaffif et al., 2025). Much like how studies of parasites demonstrated the plasticity of eukaryotic cells, expanding our repertoire of model systems promises to uncover new principles of cellular evolution that remain hidden when research is confined to a small number of genetically tractable but potentially unrepresentative organisms. Culturing new models still presents a significant bottleneck, but efforts are underway to systematise the large-scale culturing of hitherto uncultivated microbial species (Salcher et al., 2025).

## Challenge 4 – integrating ecology with evolutionary cell biology

Another area of excitement in the field is the integration of evolutionary cell biology with ecology. Ecological forces clearly shape cellular phenotypes on evolutionary timescales, but the nature of this influence depends heavily on the plasticity of the phenotype under investigation (Jarosz et al., 2014; Carja and Plotkin, 2017) as well as population dynamics and the link between ecology and individual fitness (Lynch and Trickovic, 2020). Further complicating matters, the environment is always changing, and historical ecologies are not always easily inferred. Therefore, the ecological influence on evolutionary trajectories leading up to the present day is often invisible. Thus, evolutionary cell biology, which has been largely preoccupied with deep evolution and the emergence of fundamental cellular features, has been for the most part divorced from the ecological context in which evolution took place. New tools to reconstruct and predict genomes, proteins and ecological settings, along with the need to understand cellular resilience mechanisms in the context of pathogenesis and a rapidly changing climate, mean that the status quo is changing and will change further over the course of the field's second decade.

When it has been possible to establish a direct, unambiguous link between an observable cellular phenotype and fitness in the context of a specific ecological context, it has resulted in striking insights. For example: frog-killing chytrid fungi evolved an encystation response to amphibian mucus (Robinson et al., 2022), the induction of aggregative multicellularity in the choanoflagellate *Choanoeca flexa* is an evolved response to desiccation cycles in tidal pools (Ros-Rocher et al., 2024 preprint), and the broadening of the wavelength detection range of the diatom phytochrome (DPH) has allowed marine diatoms to adapt to the amount of light in the water column (Duchêne et al., 2025). Based on these early studies, we should aim to create a framework for ‘eco–evolutionary cell biology’. Such a framework will allow our thinking to project forward in time, predicting the physiology of the cells of the future in the face of the next pandemic or dramatic shift in ocean and land surface temperatures (Chen et al., 2024; Moeller et al., 2024).

## Outlook

Over the past decade, evolutionary cell biology has moved from a loosely defined intersection of parasitology, comparative morphology and molecular genetics to an increasingly cohesive discipline. Given the inherent scale and complexity of evolutionary cell biology, collaborative initiatives (e.g. large consortia generating multi-omic data or community platforms for functional annotation) are vital to growth of the field. Equally vital is the work of smaller groups that continue to contribute deep, mechanistic studies on select model systems or neglected lineages – work that can be amplified through integration with large-scale data repositories. By adopting experimental evolution, phylogenomic and genetic approaches, and an expanded range of model systems, we can now address not only what has evolved in cellular structures and processes but also why and how such changes unfold. Integrating ecology, building new models and embracing natural diversity will undoubtedly push the field further, illuminating fundamental principles of cell evolution and potentially helping us predict how cells respond to future environmental and biological challenges.
